# FADD adaptor in cancer

**DOI:** 10.1186/1476-9433-4-1

**Published:** 2005-02-17

**Authors:** Léa Tourneur, Agnès Buzyn, Gilles Chiocchia

**Affiliations:** 1 Département d'Immunologie, Institut Cochin, INSERM U 567, CNRS UMR 8104, IFR 116, Université René Descartes, Paris V, Paris, France; 2 Service d'Hématologie Adultes, Hôpital Necker-Enfants Malades, Paris, France

## Abstract

FADD (Fas Associated protein with Death Domain) is a key adaptor molecule transmitting the death signal mediated by death receptors. In addition, this multiple functional protein is implicated in survival/proliferation and cell cycle progression. FADD functions are regulated via cellular sublocalization, protein phosphorylation, and inhibitory molecules. In the present review, we focus on the role of the FADD adaptor in cancer. Increasing evidence shows that defects in FADD protein expression are associated with tumor progression both in mice and humans. Better knowledge of the mechanisms leading to regulation of FADD functions will improve understanding of tumor growth and the immune escape mechanisms, and could open a new field for therapeutic interventions.

## The FADD molecule

The FADD gene is located on chromosome 11q13.3 in humans and 7 in mice [1]. Mutations in the FADD gene containing locus are frequently observed in human malignancies [2]. For instance, the 11q13 region contains the fibroblast growth factor 3 and 4 genes which are coamplified in melanoma. It also includes the multiple endocrine neoplasia I gene whose mutation leads to tumor development of several endocrine glands including thyroid. Moreover, two genes implicated in leukemia are found in this locus: NUMA1 which is translocated in acute promyelocytic leukemia, and BCL1 which is located very close to the FADD gene and is mutated in B-cell leukemia/lymphoma. Although FADD has a central role in multiple receptor-induced cell death as discussed hereafter, no mutation of the FADD gene itself has been reported so far.

Human and mouse FADD genes have the same quite simple organization consisting of two exons (286 bp and 341 bp in humans; 332 bp and 286 bp in mice) separated by a unique intron of approximately 2 kb. Interestingly, no cap site was reported on the human FADD mRNA [1], suggesting a particular regulation of FADD mRNA translation, although this topic has not been further investigated.

**Figure 1:**
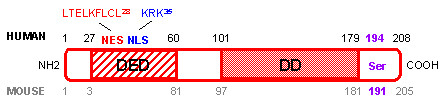
**Human and mouse FADD protein. **Amino acids (AA) corresponding to the human FADD protein are marked in black, whereas AA corresponding to the mouse FADD protein are marked in grey. The death domain (DD) and death effector domain (DED) are essential for interaction with death receptors and transmission of the apoptotic signal. Human nuclear export sequence (NES in red) and nuclear localization sequence (NLS in blue) determine localization of the protein in the cytoplasm and the nucleus, which are associated with cell death and survival functions of the FADD protein, respectively. Human Ser 194 and mouse Ser 191 phosphorylation site (in purple) have a crucial role in survival/proliferation and cell cycle progression.

Human and mouse FADD proteins are very similar (Figure 1). They consist of 208 and 205 amino acids (AA) respectively, and share 80% similarity and 68% identity [3]. FADD mRNA and protein are almost ubiquitously expressed in fetal and adult tissues, both in humans and mice [4]. Two domains are particularly well conserved between species: the death domain (DD) at the COOH-terminus of the protein, and the death effector domain (DED) at the NH2-terminus of the protein [5,6]. Both domains play a crucial role in transducing the apoptotic signal mediated by death receptors. Furthermore, a single serine (Ser) phosphorylation site essential for determining cell cycle progression is conserved in both species (human Ser 194 [7] and mouse Ser 191 [8]).

Since the first role ascribed to FADD was to transmit apoptotic signals through its interaction with death receptors expressed at the cell membrane, it was assumed that FADD protein was exclusively localized in the cytoplasm of the cell. However, a nuclear localization sequence (NLS) and a nuclear export sequence (NES) were recently identified in the human FADD protein (Figure 1), and account for FADD protein expression in the nucleus and the cytoplasm of the cell, respectively [9]. The vast majority of the reports on FADD focused on the cytoplasmic FADD protein because of its pro-apoptotic function. In contrast, the role of the nuclear FADD is much more mysterious. It was recently reported that FADD expression in the nucleus protects cells from apoptosis, but the mechanism implicated in this survival function has not been investigated [9]. On the other hand, it has been shown that FADD could interact within the nucleus of adherent cells with the methyl-CpG binding domain protein 4 (MBD4) [10]. MBD4 is a GT mismatch repairing protein. Association between MBD4 and FADD within the nucleus could couple MBD4-mediated genome surveillance with FADD-mediated cell death. Thus, nuclear FADD could perhaps have a pro-apoptotic function, at least in response to DNA-damaging agents.

## Functions of the FADD protein

### An essential molecule for embryonic development

The essential role of the FADD molecule was highlighted by generating FADD mutant null mice [11,12]. Indeed, FADD knockout mice were not viable. FADD null embryos died *in utero *at day 12.5 of development, due to underdevelopment, abdominal hemorrhage, and cardiac failure. Moreover, FADD loss of function did not result in a lymphoproliferative disorder as observed in viable Fas mutant mice [13,14]. These results indicated that in addition to its well known role in cell death, FADD was also implicated in survival/proliferation of some cell types.

### A main death transducer for DD-containing receptors

**Figure 2:**
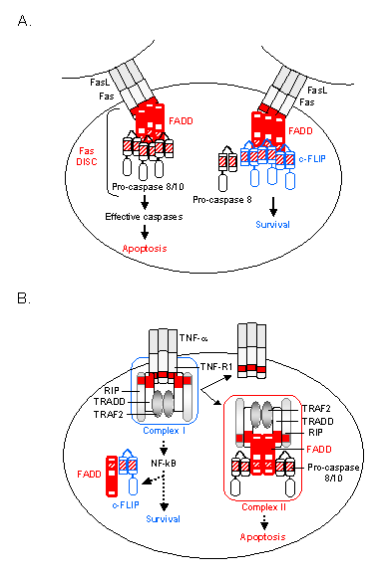
**Apoptosis mediated by death receptors requires the FADD adaptor molecule. (A) **Engagement of a Fas ligand trimer on a trimer of Fas leads to FADD adaptor molecule recruitment through homotypic DD interactions. FADD next binds initiator pro-caspase through DED interactions. This Fas/FADD/pro-caspase complex forms the Fas death-inducing signaling complex (DISC) since initiator pro-caspase activates a caspase cascade resulting in apoptotic death of the cell. Alternatively, c-FLIPs can promote cell survival by interacting with FADD through their respective DED, thus hindering recruitment and activation of initiator pro-caspase. **(B) **Signaling mediated by TNF-R1 implicates formation of two sequential complexes [61]. The complex I (in blue) contains the TNF-R1, the adaptor TRADD, the receptor interacting kinase (RIP), and the TNF-receptor associated factor 2 (TRAF2). It assemblies rapidly following TNF-α stimulation and activates the NF-kB pathway which in turn induces expression of survival genes, including c-FLIP. Later on, complex I dissociates from the TNF-R1 and is internalized. FADD can then bind the liberated DD of TRADD and recruits initiator pro-caspase, forming complex II (in red) which is cytoplasmic. Activation of initiator pro-caspase 8/10 in complex II results in apoptosis of the cell. Red box: DD; hatched red box: DED.

FADD is the main signal transducing intermediate adaptor molecule of several death receptors including Fas, TNF-R1 (tumor necrosis factor receptor 1), DR3 (death receptor 3), TRAIL-R1 (TNF-related apoptosis-inducing ligand, DR4), and TRAIL-R2 (DR5) [4,11,12,15]. All these receptors possess, in their intra-cytoplasmic tail, a DD homologous to the DD of FADD allowing FADD recruitment to the activated receptor. FADD can be recruited either directly to Fas and TRAIL-Rs (Figure 2A) or indirectly to TNF-R1 (Figure 2B). In the latter case, FADD is recruited via another DD-containing adaptor molecule (TRADD, TNF receptor-associated protein with DD). Next, FADD recruits DED-containing initiator pro-caspase 8 or 10 through DED/DED interactions [16-18], thus forming the death-inducing signaling complex (DISC) [19]. Autoprocessing of initiator pro-caspase leads to activation of effective caspases which cleave intracellular substrates, causing the apoptotic death of the cell [20,21].

Control of FADD recruitment to the DISC can occur following several mechanisms depending on the cell type and the death receptor [22]. The best characterized death receptor signaling inhibitors are DED-containing viral and cellular FLICE-inhibitory proteins (v-FLIPs and c-FLIPs, respectively) [23,24]. Inhibition of Fas-, TNF-R1-, and TRAIL-Rs-induced apoptosis by endogenous FLIPs results from binding of the c-FLIPs to the DED of FADD, thus hindering pro-caspase 8 activation (Figure 2A). Similarly, v-FLIPs inhibit apoptosis mediated by death receptors either by binding to FADD and blocking pro-caspase 8 processing, or by binding to pro-caspase 8 and inhibiting FADD interaction. Therefore, equilibrium between FADD and the expression of its inhibitors determines the outcome of the death receptor-stimulated cell, i.e. apoptosis or survival.

All the main death receptors described up to now require FADD adaptor for transmitting their apoptotic signal. Consequently, FADD is a central protein that controls multiple essential cellular processes including cellular homeostasis and elimination of pathological cells, particularly during the course of an immune response.

### Death receptor independent FADD induced apoptosis

Formation of cytoplasmic death effector filaments (DEF) by oligomerization of DED-containing proteins, including FADD, is responsible for death receptor independent cellular apoptosis [25]. Indeed, FADD over-expression by itself is known to induce cell death through DEF formation that recruits and activates pro-caspase 8. However, the existence of DEF has not been established *in vivo*, and increasing evidences suggest that DEF could be artefactual structures resulting from protein over-expression. As a consequence, the ability of endogenous FADD to aggregate and form DEF in normal situation should be reconsidered.

### Functions in proliferation and cell cycle progression

Beside being a main death adaptor molecule, FADD is also required for T cell proliferation. The first evidence of this property of FADD came from observations made in chimeric FADD knockout mice. Five-week-old chimeric FADD^-/- ^mice presented a lack of thymocytes compared to wild type animals, with few or no CD4^+ ^CD8^+ ^double positive thymocytes remaining [12]. Moreover, several groups have demonstrated that FADD deficiency in peripheral T lymphocytes resulted in an inhibition of mitogen-induced T cell proliferation [12,26-30]. The mechanism leading to FADD-dependent T cell proliferation did not involve the early events associated with cell proliferation since expression level and functionality of the IL-2 receptor, level of IL-2 secretion, mobilization of intracellular calcium, and activation of NF-kB, p38-MAPK, and p44/42-MAPK appeared normal in FADD^-/- ^T lymphocytes [12,29,30]. Recent data showed that FADD^-/- ^T lymphocytes entered the cell cycle upon mitogenic stimulation, but died during progression through the cell cycle [30]. Therefore, lack of proliferation of FADD-deficient T cells results from defective survival associated with progression through the cell cycle rather than defective activation. Up to now, the molecular pathway implicated in FADD-mediated survival of lymphocytes has not been described.

In addition to impairing survival during cell division, FADD deficiency also leads to a dysregulation of the cell cycle machinery [31]. The pattern of expression of molecules implicated in both G1/S and G2/M transitions was aberrant in FADD^-/- ^lymphocytes, resulting in spontaneous entry and progression through the cell cycle of 10% of freshly isolated FADD^-/- ^T cells (as compared to less than 2% of wild type T cells) [31].

The mechanisms responsible for FADD regulation of cell cycle progression are not fully understood. However, the phosphorylation of the 194 human and 191 mouse Ser of the protein (Figure 1) has recently drawn attention. Indeed, human FADD was phosphorylated at Ser 194, by a still unidentified 70 kDa protein kinase, in cells arrested in G2/M, whereas it was unphosphorylated in G1/S [7]. Generation of FADD Ser 191 mutant mice confirmed that FADD phosphorylation is involved in proliferation *in vivo *[8]. Replacement of Ser 191 by an aspartic acid resulting in constitutive phosphorylation of the FADD protein led to abnormal development of FADD mutant mice that shared the same phenotype as FADD deficient mice [8], including few CD4^+ ^CD8^+ ^thymocytes and defective progression through the cell cycle [12,31].

Tumor cells are constantly cycling cells. Although it does not seem to directly affect the cell cycle progression, FADD phosphorylation at Ser 194 sensitizes these cells to reagents that induce G2/M arrest such as the Taxol anticancer drug [32]. In human prostate cancer cell lines, treatment with Taxol resulted in Ser 194 FADD phosphorylation and G2/M arrest [33]. Moreover, etoposide or cisplatin chemotherapeutic drug-induced apoptosis of these cells was enhanced by pretreatment with Taxol, a process that was inhibited by cellular over-expression of an unphosphorylable FADD mutant [33]. Therefore, tumor cells that express a Ser 194 FADD mutant that cannot be phosphorylated or are unable to phosphorylate FADD at this Ser position are expected to resist apoptosis induced by anticancer drugs that induce G2/M arrest, and to be insensitive to the synergistic effect of chemotherapy. Obviously, a lack of FADD expression will have the same consequences.

## Role in tumor development

### FADD as a tumor suppressor

The role of the FADD adaptor in cancer was initially demonstrated by generating RAG-1 deficient transgenic mice that target expression of a FADD dominant negative mutant in lymphocytes. With age these mice developed thymic lymphoblastic lymphoma, whereas FADD^+/+ ^RAG-1^-/- ^mice did not [34]. Moreover, thymic lymphoblastic lymphomas were never observed in FADD^-/- ^RAG-1^+/+ ^or FADD^-/- ^RAG-1^+/- ^mice, demonstrating that absence of FADD expression was necessary but not sufficient to induce tumor development in this model [34]. These results were the first demonstration that FADD adaptor can act as a tumor suppressor *in vivo*.

Using a mouse model of thyroid adenoma/adenocarcinoma, we showed spontaneous disappearance of FADD protein expression during the course of tumor development [35]. The so called gsp transgenic mice expressed an oncogene specifically in thyroid follicular cells (TFC), and developed thyroid hyperplasia that eventually transformed into hyperfunctioning adenomas or adenocarcinomas around the age of 8 months [36]. The fact that gsp mice developed hyperfunctioning adenomas or adenocarcinomas belatedly suggested that oncogene expression conferred a predisposition but that an additional event was necessary for thyroid tumor development. We found that FADD protein was highly expressed in all non-pathological and in almost all hyperplastic thyroid glands from gsp mice. In contrast, thyroid adenoma/adenocarcinoma expressed low or no FADD protein [35]. These results raised the possibility that loss of FADD protein expression could be an additional event contributing to tumorigenesis, and suggested that FADD plays a role as a tumor suppressor.

We recently showed that absence of FADD protein expression in cancer cells is also a relevant phenomenon in human malignancies [37]. We looked for FADD protein expression in human acute myeloid leukemia (AML) cells. Leukemic cells of most AML patients are resistant to Fas-mediated cell death despite expressing the Fas receptor [38] and/or the FasL molecule [39]. Moreover, chemotherapeutic drugs used for AML treatment can kill target cells via several mechanisms, including death receptor-induced apoptosis [40-43]. We performed a retrospective study of 70 consecutive patients with *de novo *AML treated homogeneously, and found that leukemic cells of 2/3 of patients at diagnosis expressed low or no FADD protein [37]. Moreover, in this cohort of patients, we showed that absent/low FADD protein expression in AML cells at diagnosis was a new independent prognostic factor for poor response to chemotherapy (in terms of complete remission rate, event free and overall survivals) [37]. Importantly, absent/low FADD protein expression in AML cells at diagnosis was a prognostic factor even for patients classified as standard- or good-risk AML cases by cytogenetic and molecular criteria [37]. As a consequence, this new prognostic factor is of clinical importance since it will allow early identification of patients with chemoresistant AML who could benefit from more intensive post-remission therapy.

### Absence of FADD confers numerous advantages on cancer cells

**Figure 3:**
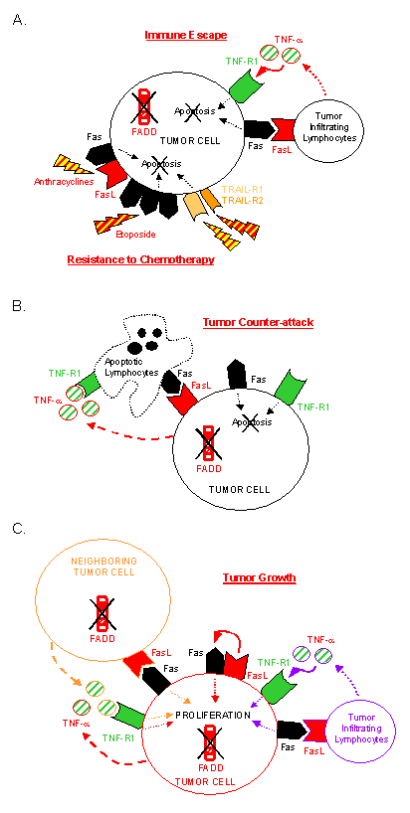
**Lack of FADD expression confers survival/growth advantages on tumor cells. **Absence of FADD protein confers multiple death receptor-mediated apoptosis resistance and allows tumor cells to co-express death receptors and ligands without committing cell death. **(A) **Lack of FADD contributes to immune escape and resistance to chemotherapy. FADD deficient tumor cells resist death receptor-mediated apoptosis induced by TIL and chemotherapeutic drugs. Anthracyclines increase Fas and FasL expression on tumor cells. Etoposide induces Fas receptor trimerization, leading to Fas-mediated cell death independently of FasL expression. Both drugs enhance TRAIL-R2-mediated apoptosis. **(B) **Lack of FADD contributes to tumor counter-attack. Lack of FADD expression allows many types of tumor cells to express innocuously functional FasL that can kill TIL. Secretion of TNF-α by AML cells can have cytotoxic effects on TIL. **(C) **Lack of FADD contributes to tumor growth. In the absence of FADD, Fas signaling leads to a proliferative signal instead of an apoptotic one. Concomitant death receptor and ligand expression, in the absence of FADD, allows autocrine (in red) and paracrine (in orange) proliferation of tumor cells. Activated TIL can contribute to paracrine (in purple) proliferation of FADD-deficient tumor cells.

The fact that absence of FADD expression was found in different types of tumor cells both in mice and humans strongly suggested that absence of FADD contributed to tumor development. Indeed, lack of FADD protein can confer numerous advantages on pathological cells, which predominantly result in tumor survival/growth gain (Figure 3).

#### Immune escape and resistance to chemotherapy

Fas, TRAIL-Rs, TNF-R1, DR3, and potentially other receptors use FADD adaptor for transmitting the death signal. Thus, absence of FADD expression in tumor cells must confer multiple resistance of these cells to death receptor cytotoxicity. In agreement with this assumption, we observed that FADD^-/- ^TFC as well as FADD^-/- ^AML cells were resistant to Fas- and TNF-α-mediated apoptosis [35], and unpublished data). Since cytotoxic tumor infiltrating lymphocytes (TIL) use, among other mechanisms, death receptor-mediated cell death to kill pathological cells, tumor cells lacking FADD molecule expression may partially avoid immune attack (Figure 3A). On the other hand, some anticancer drugs exert their cytotoxic effect by inducing death receptor and/or death ligand expression on tumor cells, thereby inducing suicidal/fratricidal apoptosis of the cells. For instance, anthracyclines and etoposide, two chemotherapeutic drugs used for AML treatment, enhance Fas- and TRAIL-R2-mediated cell death *in vitro*, a process requiring FADD molecule expression [40-42,44,45]. As a consequence, absence of FADD expression in AML cells of our patients may contribute to chemoresistance of leukemic cells (Figure 3A).

#### Tumor counter-attack

As described above, absence of FADD allows co-expression of death receptors and ligands without inducing autocrine or paracrine apoptosis of tumor cells. For instance, although the Fas receptor was expressed at all stages of thyroid tumor development in gsp mice, FasL expression was gained with a high expression in adenomatous/adenocarcinomatous glands [35]. Using the same method, we found that leukemic cells of most AML patients expressed FasL [39] and secreted TNF-α despite expressing Fas and TNF-R1 [37], and unpublished data). Moreover, co-expression of Fas and FasL molecules that did not cause cell death was also observed in lymphoma [46], melanoma [47], astrocytoma [48], cancers of the colon [49], liver [50], lung [51], human thyroid [52]. The role of death ligand expression in tumor cells is still a controversial issue [53-55], but it is now well accepted that it allows at least some cancer cells to kill TIL that express death receptors, a process called the "tumor counter-attack" (Figure 3B).

#### Proliferative advantage

Since the tumor counter-attack hypothesis cannot apply to all types of cancer cells, one could wonder whether other benefits of death ligand expression by tumor cells exists. Previous reports demonstrated that Fas signaling could lead to proliferation instead of apoptosis, depending on the cell type and the environmental conditions [56-58]. For instance, agonistic anti-Fas antibody-induced proliferation of hematological and non-hematological tumors has been described [59]. Moreover, we have shown that stimulation of FADD lacking thyrocytes by an agonistic anti-Fas antibody resulted in accelerated growth of TFC, via a particular Daxx adaptor-mediated pathway [35]. Therefore, Fas signaling, particularly in the absence of FADD, can confer proliferative advantage on tumor cells (Figure 3C). Thus, FasL expression on Fas^+ ^FADD^- ^adenomatous/adenocarcinomatous thyroid from gsp mice, as well as on human AML cells, may allow both autocrine and paracrine proliferation of these cells [35,37] (Figure 3C). Furthermore, we can formulate the same hypothesis for TNF-α secretion by TNF-R1 expressing leukemic cells (Figure 3C). If our hypotheses are correct, then activated TIL that express death ligands could contribute to FADD-deficient tumor proliferation (Figure 3C).

### Restoring FADD protein expression- a new therapeutic issue

Absence of FADD expression could confer multiple growth advantages on cancer cells (Figure 3), and is expected to contribute to disease progression. As a consequence, finding how to restore FADD protein expression in FADD-negative tumor cells represents a research field with potentially direct clinical applications. In fact, it is possible that some of the current cancer treatments act, at least partially, through this mechanism. For instance, carboplatin is a cytotoxic drug potentially effective at reestablishing functional FADD protein expression. Indeed, the human tongue carcinoma cell lines SCC-9 and SCC-25 express very low levels of FADD protein. Moreover, treatment with carboplatin enhances FADD protein expression, thus rendering cancer cells sensitive to Fas-mediated apoptosis [60]. The combination of carboplatin with chemotherapeutic drugs that induce death receptor-mediated cell death may result in improved treatment.

Molecules implicated in FADD regulation of expression also represent new therapeutic targets. However, very little is known about the mechanisms leading to absent/low FADD protein expression in tumor cells. In SCC-9 and SCC-25 carcinoma cell lines, carboplatin upregulated FADD protein expression by increasing FADD mRNA [60]. However, FADD mRNA was normally expressed in mouse thyroid adenoma/adenocarcinoma and in human AML cells, and lack of FADD mRNA could not account for poor FADD protein expression in these cancer cells [35,37]. These results suggest that several mechanisms could be implicated in loss of FADD protein, depending on the type of cell and the environmental pressure. Besides, docking FADD protein away from the death receptor, in the nucleus for example, would have the same consequences. Understanding such mechanisms is the first necessary step towards development of new anticancer drugs targeting molecules that regulate FADD protein expression.

## Conclusion

FADD is mainly known as a key adaptor molecule for numerous death receptors. However, increasing evidences have shown that FADD is a much more complex molecule implicated in apoptosis, survival, cell cycle progression, and proliferation of the cells. Therefore, FADD plays a central role in the frightening control of cell death and life. As a consequence, a defect in the FADD molecule can contribute to the development of diseases, and particularly cancer. Absence of FADD protein expression is a marker of tumor development in the mouse, and a prognostic factor for poor response to chemotherapy in humans. Since FADD deficiency could contribute to several malignancies, in view of its almost ubiquitous pattern of expression, study of the role of FADD in tumor development, growth, and resistance to treatment, and understanding how the expression of this puzzling molecule is regulated, are targets that merit further investigation.

## Authors' Contributions

LT conceived the review and drafted the manuscript. GC participated in conceiving the review. AB and GC participated in the preparation of the manuscript. All authors read and approved the final manuscript.
